# Supplementary material for: End Sequence Analysis Toolkit (ESAT) expands the extractable information from single-cell RNA-seq data
Source: Genome Res. 2016 Oct;26(10):1397–410. doi: 10.1101/gr.207902.116 (PMC5052061; doi:10.1101/gr.207902.116)
Supplement: Supplemental Material [file supp_gr.207902.116_Supp_Materials.tar.gz › ESAT-esat_v0.1/doc/html/classumms_1_1core_1_1utils_1_1_annotation_utils.html]

ESAT: umms.core.utils.AnnotationUtils Class Reference


ESAT


- Main Page
- Related Pages
- Packages
- Classes
- Files

- Class List
- Class Index
- Class Hierarchy
- Class Members

All Classes Namespaces Files Functions Variables Enumerator Pages

- umms
- core
- utils
- AnnotationUtils

Static Public Member Functions |
Static Public Attributes |
List of all members

umms.core.utils.AnnotationUtils Class Reference

Collaboration diagram for umms.core.utils.AnnotationUtils:

[legend]

|  |  |
| --- | --- |
| Static Public Member Functions | |
| static Map< String, Collection  < Gene > > | collapseOverlappers (Map< String, Collection< Gene >> genes, boolean ignoreStrand) |
|  | |
| static boolean | pairwiseNonoverlappingIgnoreStrand (Collection< Annotation > regions) |
|  | |
| static Collection< Annotation > | mergeOverlappingBlocks (TreeSet<?extends Annotation > windows) |
|  | |
| static< TextendsAnnotation >  Collection< Annotation > | mergeOverlappingBlocksSingleOrientation (Collection< T > regions, boolean enforceSameOrientation) |
|  | |
| static< TextendsAnnotation > T | getLargestParent (T child, Map< String, Collection< T >> parents) |
|  | |
| static< TextendsAnnotation >  Map< T, T > | mapChildToLargestParent (Map< String, Collection< T >> children, Map< String, Collection< T >> parents) |
|  | |
| static< TextendsAnnotation >  Collection< T > | getParents (T child, Map< String, Collection< T >> parents) |
|  | |
| static< TextendsAnnotation >  Collection< T > | getChildren (T parent, Map< String, Collection< T >> children) |
|  | |
| static< TextendsAnnotation >  Map< T, Collection< T > > | mapParentsToChildren (Map< String, Collection< T >> children, Map< String, Collection< T >> parents) |
|  | |
| static< TextendsAnnotation >  Map< T, Collection< T > > | mapChildToParents (Map< String, Collection< T >> children, Map< String, Collection< T >> parents) |
|  | |
| static Map< String, Collection  < Annotation > > | loadAnnotations (String bedFile) throws IOException |
|  | |
| static int | getMidpoint (Annotation annot) |
|  | |
| static int | getSubAnnotationMidpointWithinAnnotation (Annotation annot, Annotation subAnnot) |
|  | |

|  |  |
| --- | --- |
| Static Public Attributes | |
| static Logger | logger = Logger.getLogger(AnnotationUtils.class.getName()) |
|  | |

## Detailed Description

Author
:   prussell

## Member Function Documentation

|  |  |  |  |
| --- | --- | --- | --- |
| static Map<String, Collection<Gene> > umms.core.utils.AnnotationUtils.collapseOverlappers | ( | Map< String, Collection< Gene >> | *genes*, |
|  |  | boolean | *ignoreStrand* |
|  | ) |  |  |

static

Here is the caller graph for this function:

|  |  |  |  |
| --- | --- | --- | --- |
| static <TextendsAnnotation> Collection<T> umms.core.utils.AnnotationUtils.getChildren | ( | T | *parent*, |
|  |  | Map< String, Collection< T >> | *children* |
|  | ) |  |  |

static

Get children of parent annotation

Parameters
:   |  |  |
    | --- | --- |
    | parent | Parent |
    | children | Potential children by chromosome |

Returns
:   Set of children contained in parent or empty set if none

Here is the caller graph for this function:

|  |  |  |  |
| --- | --- | --- | --- |
| static <TextendsAnnotation> T umms.core.utils.AnnotationUtils.getLargestParent | ( | T | *child*, |
|  |  | Map< String, Collection< T >> | *parents* |
|  | ) |  |  |

static

Get largest parent

Parameters
:   |  |  |
    | --- | --- |
    | child | Child annotation |
    | parents | Possible parent annotations by chromosome |

Returns
:   Largest parent (annotation containing child on same strand) or null if none exists

Here is the call graph for this function:

Here is the caller graph for this function:

|  |  |  |  |  |  |
| --- | --- | --- | --- | --- | --- |
| static int umms.core.utils.AnnotationUtils.getMidpoint | ( | Annotation | *annot* | ) |  |

static

Get midpoint along mature transcript of a spliced annotation

Parameters
:   |  |  |
    | --- | --- |
    | annot | Annotation |

Returns
:   Genome coordinate of mature transcript midpoint

|  |  |  |  |
| --- | --- | --- | --- |
| static <TextendsAnnotation> Collection<T> umms.core.utils.AnnotationUtils.getParents | ( | T | *child*, |
|  |  | Map< String, Collection< T >> | *parents* |
|  | ) |  |  |

static

Get parents of child annotation

Parameters
:   |  |  |
    | --- | --- |
    | child | Child |
    | parents | Parents by chromosome |

Returns
:   Set of parents containing child or empty set if none

Here is the caller graph for this function:

|  |  |  |  |
| --- | --- | --- | --- |
| static int umms.core.utils.AnnotationUtils.getSubAnnotationMidpointWithinAnnotation | ( | Annotation | *annot*, |
|  |  | Annotation | *subAnnot* |
|  | ) |  |  |

static

Get midpoint of a sub annotation with respect to a possibly spliced parent annotation Ignores blocks of sub annotation and only considers blocks of parent annotation

Parameters
:   |  |  |
    | --- | --- |
    | annot | Parent annotation |
    | subAnnot | Sub annotation contained in parent annotation |

Returns
:   Midpoint of sub annotation span considering blocks of parent annotation

Here is the call graph for this function:

|  |  |  |  |  |  |
| --- | --- | --- | --- | --- | --- |
| static Map<String, Collection<Annotation> > umms.core.utils.AnnotationUtils.loadAnnotations | ( | String | *bedFile* | ) | throws IOException |

static

Load annotations in the bed file as annotation objects

Parameters
:   |  |  |
    | --- | --- |
    | bedFile | Bed file |

Returns
:   Map of chromosome to collection of annotations

Exceptions
:   |  |  |
    | --- | --- |
    | IOException |  |

|  |  |  |  |
| --- | --- | --- | --- |
| static <TextendsAnnotation> Map<T, T> umms.core.utils.AnnotationUtils.mapChildToLargestParent | ( | Map< String, Collection< T >> | *children*, |
|  |  | Map< String, Collection< T >> | *parents* |
|  | ) |  |  |

static

Map each child annotation to its largest parent defined as annotations overlapping and containing child

Parameters
:   |  |  |
    | --- | --- |
    | children | Child annotations by chromosome |
    | parents | Parent annotations by chromosome |

Returns
:   Map of child to largest parent. Does not include children with no parents.

Here is the call graph for this function:

|  |  |  |  |
| --- | --- | --- | --- |
| static <TextendsAnnotation> Map<T, Collection<T> > umms.core.utils.AnnotationUtils.mapChildToParents | ( | Map< String, Collection< T >> | *children*, |
|  |  | Map< String, Collection< T >> | *parents* |
|  | ) |  |  |

static

Map each child annotation to its set of parents defined as annotations overlapping and containing child

Parameters
:   |  |  |
    | --- | --- |
    | <T> |  |
    | children | Child annotations by chromosome |
    | parents | Parent annotations by chromosome |

Returns
:   Map of child to set of parents. Does not include children with no parents.

Here is the call graph for this function:

|  |  |  |  |
| --- | --- | --- | --- |
| static <TextendsAnnotation> Map<T, Collection<T> > umms.core.utils.AnnotationUtils.mapParentsToChildren | ( | Map< String, Collection< T >> | *children*, |
|  |  | Map< String, Collection< T >> | *parents* |
|  | ) |  |  |

static

Map each parent annotation to its set of contained children

Parameters
:   |  |  |
    | --- | --- |
    | <T> |  |
    | children | Child annotations by chromosome |
    | parents | Parent annotations by chromosome |

Returns
:   Map of parent to set of children. Annotations with no children map to empty set.

Here is the call graph for this function:

|  |  |  |  |  |  |
| --- | --- | --- | --- | --- | --- |
| static Collection<Annotation> umms.core.utils.AnnotationUtils.mergeOverlappingBlocks | ( | TreeSet<?extends Annotation > | *windows* | ) |  |

static

Merge annotations that overlap in the same orientation as each other, and leave singleton windows the same

Parameters
:   |  |  |
    | --- | --- |
    | windows | Annotation set |

Returns
:   New set of merged windows

Here is the call graph for this function:

|  |  |  |  |
| --- | --- | --- | --- |
| static <TextendsAnnotation> Collection<Annotation> umms.core.utils.AnnotationUtils.mergeOverlappingBlocksSingleOrientation | ( | Collection< T > | *regions*, |
|  |  | boolean | *enforceSameOrientation* |
|  | ) |  |  |

static

Merge annotations that overlap, and leave singleton windows the same All returned annotations will have the same orientation: unknown if not enforcing same orientation, or the common orientation of all input regions if enforcing

Parameters
:   |  |  |
    | --- | --- |
    | regions | Annotation set |
    | enforceSameOrientation | Require that all regions have same orientation to begin with |

Returns
:   New set of merged windows

Here is the call graph for this function:

Here is the caller graph for this function:

|  |  |  |  |  |  |
| --- | --- | --- | --- | --- | --- |
| static boolean umms.core.utils.AnnotationUtils.pairwiseNonoverlappingIgnoreStrand | ( | Collection< Annotation > | *regions* | ) |  |

static

Check if the entire set is pairwise non-overlapping, ignoring strand

Parameters
:   |  |  |
    | --- | --- |
    | regions | Set of regions |

Returns
:   True if no blocks overlap

Here is the call graph for this function:

## Member Data Documentation

Logger umms.core.utils.AnnotationUtils.logger = Logger.getLogger(AnnotationUtils.class.getName())

static

---

The documentation for this class was generated from the following file:

- java/umms/core/utils/AnnotationUtils.java


---

Generated on Fri Aug 15 2014 15:11:15 for ESAT by  

 1.8.7
